# The associations between coping strategies, psychological health, and career indecision among medical students: a cross-sectional study in China

**DOI:** 10.1186/s12909-021-02781-x

**Published:** 2021-06-09

**Authors:** Yaxin Zhu, Tianming Zuo, Yanni Lai, Shenglin Zhao, Bo Qu

**Affiliations:** 1 Institute for International Health Professions Education and Research, China Medical University, No. 77 Puhe Road, Shenyang North New Area, Shenyang, Liaoning P.R. China 110122; 2 Medical Education Office, Fudan University, No. 220 Handan Road, Yangpu District, Shanghai, P.R. China 200433; 3 School of Public Health, China Medical University, No. 77 Puhe Road, Shenyang North New Area, Shenyang, Liaoning P.R. China 110122

**Keywords:** Career indecision, Medical students, Coping strategies, Psychological health

## Abstract

**Background:**

Medical students experience difficulties in the process of making decisions about their careers, which is referred to as career indecision. This study aimed to examine the difficulties in the career decision-making processes of medical students and to explore the association of coping strategies and psychological health with career indecision. The findings may provide a reference for designing interventions to advance satisfying career decisions for medical students.

**Methods:**

A cross-sectional survey of 359 medical students was conducted in 5 Chinese medical schools. Students completed an anonymous self-administered questionnaire measuring their career indecision, coping strategies, and psychological health. Independent t-test, F-test, bivariate Pearson’s correlation analysis, and linear regression analysis were applied to test the relation between career indecision and the associated factors. Data were analyzed using SPSS V.22 for Windows. A *p*-value < 0.05 was considered to be statistically significant.

**Results:**

Difficulties regarding lack of readiness frequently occurred in medical students when making career decisions, with the highest score of 2.48 ± 0.58. Among all the associated factors in this study, career indecision was positively associated with psychological distress problem (β = 0.20, *p* < 0.05). This study also proved that being at a higher level of career indecision is negatively associated with using problem-focused coping strategies (β = − 0.14, *p* < 0.05). For the maladaptive coping strategies, applying dysfunctional coping strategies showed a significantly positive association with career indecision among medical students (β = 0.25, *p* < 0.05).

**Conclusions:**

Medical students experienced difficulties regarding lack of readiness frequently when making career decisions. Both coping strategies and psychological health were associated with career indecision among medical students. To prevent career indecision, it is necessary to promote earlier career awareness to medical students. Specifically, psychological health should be addressed in career intervention programs for medical students. Additionally, when helping medical students to cope with career indecision, cognitive techniques that reduce the use of maladaptive coping strategies and enhance the use of adaptive coping strategies should be adopted.

## Background

Career indecision describes the difficulties people may experience in the process of making decisions about their careers [1]. It has been considered to be a multidimensional construct with various subtypes of difficulties during the career decision-making process. The widely applied construct framework covered 3 groups of difficulties as follows: Lack of Readiness (LR), Lack of Information (LI), and Inconsistent Information (II). LR occurred before one began the process of making career decision, and the remaining 2 groups of difficulties often arouse after the process began [2]. Career indecision was reported as particularly salient for college students, and who often seek professional help to resolve it [3].

For medical students, making decisions about their career choices is also a significant stressor [4, 5]. Considering the global shortage and unequal distribution of physicians, medical students making an appropriate decision about their career specialty could be vital not only for the medical profession but also for society as a whole [6, 7]. In recent years, some Chinese medical graduates have been unemployed [8]. The employment rate of medical students ranked ninth to last in all the major specialties across China and has dropped from 89.3 to 88.0% in the past 3 years [8]. These students may be unable to start building a professional pathway due to be blocked in their career decision-making processes [3, 9]. In China, Ministry of Education has suggested that colleges should integrate career development programs into formal curricula [10]. However, there exist some problems in the course such as lacking of self-assessment on career-related characteristics, lacking of career development practice, and offered before graduation not throughout the whole higher education process [11, 12]. A better understanding of career indecision and its associated factors will provide a reference for designing interventions to alleviate career decision-making difficulties and advance satisfying career decisions for medical students [9, 13]. However, few recent studies have focused on career indecision among medical students [14, 15].

There are various factors identified to influence career indecision among young students by previous studies, such as self-efficacy, coping style, and anxiety [1, 3, 6, 9]. The current study focused on the influences of coping and psychological distress on career indecision, which has recently received an increased emphasis in career indecision-related researches. Coping was defined as the cognitive attempts and behavioral adaption to deal with stressors. When encountering various difficulties, individuals may adopt different coping strategies which can be divided into two groups: adaptive and maladaptive [16, 17]. Studies have focused on the association of adaptive coping strategies and career indecision among college students; those who adopted adaptive coping strategies, such as problem-focused coping strategies, had lower level of career indecision [1, 18]. Individuals with adaptive coping strategies that inspired them to solve problems directly would tend to pursue more information and continue the path of career seeking [19]. Besides adaptive coping strategies, little is known about the association of maladaptive coping strategies with career indecision.

Psychological health status was also found to be a critical in the process of career decision-making [9]. Poorer psychological health predicted higher levels of career indecision [3, 9]. Although the association of coping and psychological health status with career indecision have separately been examined in previous research, their co-association effect on career indecision has not been explored. Taken as a whole, coping and psychological distress might mutually amplify the career indecision.

Using a reliable tool to identify specific difficulties hindering students from making career decision was important in career development program [2]. The Career Decision-making Difficulty Questionnaire (CDDQ) developed by Gati et al. is a widely used instrument for assessing career indecision in various cultural contexts [2, 20]. The Chinese version was cross-culturally adapted by Li [21]. The CDDQ is a multidimensional instrument to assess three salient facets of the career indecision construct, including LR, LI, and II.

For these reasons, two objectives have guided the research in this study. The first objective was to determine what major difficulties impair the career decision-making process among medical students, as measured by CDDQ. The second objective was to explore the association of coping strategies and psychological health with career indecision. For this second objective, the following specifical hypotheses were proposed:

- Hypothesis 1. Adaptive coping strategies will be significantly negatively associated with career indecision among medical students.
- Hypothesis 2. Maladaptive coping strategies will be significantly positively associated with career indecision among medical students.
- Hypothesis 3. Psychological distress problem will be significantly positively associated with career indecision among medical students.

## Methods

### Ethics statement

This study was approved by the ethics committee of China Medical University. The participants were informed about the purpose of the study and, prior to the start of the study, were assured that their privacy would be protected. An anonymous self-administered questionnaire was completed by students voluntarily. Written informed consent was obtained from all participants. All methods in the current study were carried out in accordance with relevant guidelines and regulations.

### Study design

A cross-sectional questionnaire survey was conducted between January and June 2019, which is the last half of the academic year. The data were analyzed using a quantitative methodology to examine the factors associated with career indecision.

### Participants

The Chinese medical education program is a “5 + 3” model, which includes 5 years of undergraduate clinical medicine studies in college (the last year was for a clerkship in hospitals) and an additional 3 years of postgraduate apprentice training as a resident. In this study, medical students were recruited from 5 medical schools located in the northern, central, and southern regions of China. A stratified clustered random sampling method was applied to select from 2 or 3 classes of students for each grade. In total, 375 medical students from the graduating and residents training stage were enrolled in this study. After excluding students with missing item responses (> 20% of the questionnaire), 359 students remained in the data set (completion rate: 95.7%).

### Measures

The survey asked the participants about their basic characteristics (Table 1) and perceptions of their specialty (Table 1). Participants also completed the CDDQ and Brief Coping Orientation to Problems Experienced (Brief COPE). Psychological health was measured by the 12-item General Health Questionnaire (GHQ-12). Information about the perception of their specialty was collected using a self-designed question asking, “Have you ever had career development experience,” which participants answered in a “yes or no” format. The Chinese version of CDDQ, Brief COPE, and GHQ-12 have been used in other studies on college students showing acceptable reliability and validity [22–24].

**Table 1:** The basic characteristics, perceptions of specialty, psychological health and career indecision among medical students (*n* = 359)

Variables	n (%)	CDDQ	LR	LI	II
**Total participants**	359 (NA)	2.42 ± 0.56	2.48 ± 0.58	2.38 ± 0.61	2.37 ± 0.66
**Basic characteristics**					
Sex^a^					
Male	150 (41.8%)	2.36 ± 0.60	2.40 ± 0.60*	2.33 ± 0.64	2.34 ± 0.71
Female	209 (58.2%)	2.46 ± 0.54	2.54 ± 0.56	2.43 ± 0.59	2.40 ± 0.63
Age^b^					
20–22	54 (15.0%)	2.52 ± 0.53	2.56 ± 0.55	2.49 ± 0.59	2.40 ± 0.66
23–25	178 (49.6%)	2.45 ± 0.54	2.51 ± 0.55	2.42 ± 0.58	2.41 ± 0.65
≥ 26	127 (35.4%)	2.34 ± 0.61	2.41 ± 0.63	2.30 ± 0.65	2.32 ± 0.68
Registered residence^a^					
Urban	162 (45.1%)	2.40 ± 0.58	2.45 ± 0.59	2.37 ± 0.62	2.34 ± 0.68
Rural	197 (54.9%)	2.44 ± 0.55	2.51 ± 0.58	2.40 ± 0.60	2.40 ± 0.66
Study year^b^					
Graduating class	115 (32.0%)	2.51 ± 0.52	2.55 ± 0.53	2.47 ± 0.58	2.48 ± 0.63
PGY-1	122 (34.0%)	2.40 ± 0.59	2.48 ± 0.60	2.40 ± 0.63	2.33 ± 0.70
PGY-2	61 (17.0%)	2.34 ± 0.58	2.43 ± 0.62	2.27 ± 0.59	2.32 ± 0.68
PGY-3	61 (17.0%)	2.35 ± 0.56	2.41 ± 0.59	2.33 ± 0.61	2.30 ± 0.63
**Perceptions of specialty**					
Experience in a career development program^a^					
Yes	131 (36.5%)	2.31 ± 0.48*	2.40 ± 0.52	2.24 ± 0.51*	2.26 ± 0.60*
No	228 (63.5%)	2.48 ± 0.60	2.53 ± 0.61	2.47 ± 0.65	2.44 ± 0.69
**Psychological health**					
GHQ ≥ 4	99 (28.0%)	2.68 ± 0.51*	2.75 ± 0.52*	2.67 ± 0.56*	2.63 ± 0.64*
GHQ<4	260 (72.0%)	2.31 ± 0.55	2.38 ± 0.57	2.28 ± 0.59	2.28 ± 0.65

*CDDQ* Career Decision-making Difficulty Questionnaire, *LR* Lack of Readiness, *LI* Lack of Information, *II* Inconsistent Information

^a^Independent t-test; ^b^F-test; *p < 0.05

The CDDQ used in this study was a 35-item instrument with a 5-point Likert scale (1-does not describe me, 5-describes me well) designed to assess the degree to which each statement describes the difficulties encountered in the process of career decision-making [21]. The difficulties were categorized into three dimensions covering LR, LI, and II. The scale and each dimension’s scores were based on averaging the item scores, ranging from 1 to 5, with a higher score indicating a stronger level of career indecision. This instrument’s internal consistency reliability coefficient in our study was 0.93, and the Cronbach’s alpha of each dimension ranged from 0.71–0.85.

The Brief COPE scale consisted of 28 items to measure 14 different coping strategies grouped into three subscales: problem-focused coping strategies (PFC, practical steps to remove or reduce the stressor), emotion-focused coping strategies (EFC, managing one’s emotional responses to stress), and dysfunctional coping strategies (DC, preventing active coping and disengaging from the stressful situation) [17]. The first two were adaptive coping strategies, and the rest were considered maladaptive. Participants were asked to score each strategy from 1 (not doing it at all) to 4 (doing it a lot). Each subscale’s scores were averaged across the item scores, ranging from 1 to 4. Strategies with higher scores indicated that participants were more likely to use these strategies. This scale presented good internal reliability in this study, with Cronbach’s alpha equal to 0.79. And the Cronbach’s alpha of each subscale ranged from 0.67–0.75.

The GHQ-12 was used to assess psychological health in this study. All 12 items were rated on a 4-point scale. The most common binary scoring method was applied, such that the responses of “less than usual” and “no more than usual” were scored equally at 0 points, and “rather more than usual” and “much more than usual” were both scored as 1 point [24]. All item scores were summed to determine the final GHQ-12 score. A score greater than or equal to 4 was considered indicative of a psychological problem. GHQ-12 has been applied among Chinese medical students [24], and the Cronbach’s alpha for this scale in this study was 0.72.

### Data analysis

Independent t-test, F-test, bivariate Pearson’s correlation analysis, and linear regression analysis were performed to test our hypotheses. In the linear regression model, the CDDQ scale and three dimensions’ scores were the dependent variables, and the enter procedure was adopted to evaluate the associated factors of career indecision after adjusting for basic characteristics, including age, sex, registered residence, and study year. In block 1, the perception of specialty was included in the model. Psychological health was added in block 2. In block 3, three kinds of coping strategies were added. Variances of career indecision explained by different groups of independent variables were examined by △*R*^2^ (R squared change). Given that variable independence is a vital precondition for the application of linear regression, the collinearity diagnostic test was carried out. The results showed that a multicollinearity problem was not present between the variables, with all the variance inflation factor (VIF) values less than 2. Data were analyzed using SPSS V.22 for Windows. A *p*-value < 0.05 was considered to be statistically significant.

## Results

### Career indecision

The CDDQ score of the overall sample was 2.42 ± 0.56 (mean ± standard deviation). For the three dimensions of CDDQ, LR had the highest score of 2.48 ± 0.58, and II showed the lowest score of 2.37 ± 0.66. The score of the LI dimension was 2.38 ± 0.61.

### Basic characteristics

A total of 150 male (41.8%) and 209 female (58.2%) students participated in the study. Their mean age was 24.99 ± 2.70 years. One hundred ninety-seven of the participants (54.9%) were from rural areas. Approximately a third of the participants (115, 32%) were graduating students. The t-test revealed that there was a significant difference in the LR score between males and females (*p* < 0.05), with female students showing higher LR scores (Table 1).

### Specialty perception characteristics

In this study, 131 medical students had experienced a career development program. Significant differences were found for the CDDQ, LI, and II scores for students with different career development experiences (*p* < 0.05), such that students with career development experience showed lower scores. The relation between specialty perception and career indecision is listed in Table 1.

### Psychological health

In this study sample, 99 medical students (28.0%) had psychological problems assessed by the GHQ-12 score being greater than or equal to 4. Significant differences were found for the scores of CDDQ and all three dimensions between students of differing psychological health status (*p* < 0.05). Medical students with psychological problems had higher career indecision scores compared to students without a psychological health problem (Table 1).

### Coping strategies

The results showed that the most common coping strategy used when medical students deal with a stressor was PFC, with the highest score of 3.41 ± 0.44. The scores of EFC and DC were 2.78 ± 0.39 and 2.22 ± 0.34, respectively. The correlation analysis showed that PFC was significantly and negatively associated with CDDQ, LI, and II score, with the correlation coefficient ranging from − 0.23 to − 0.18 (*p* < 0.05). The EFC was only significantly associated with the LI score (*r* = − 0.15, *p* < 0.05). The DC was significantly and positively associated with CDDQ and the scores for all three dimensions (*r* = 0.14–0.24, *p* < 0.05). The results are displayed in Table 2.

**Table 2:** The correlation between career indecision and coping strategies among medical students (*n* = 359)

	r between coping strategies and career indecision			
Variables	CDDQ	LR	LI	II
**PFC**	−0.18*	−0.10	− 0.23*	−0.16*
**EFC**	−0.10	−0.04	− 0.15*	−0.07
**DC**	0.23*	0.24*	0.14*	0.24*

*r* correlation coefficient, *CDDQ* Career Decision-making Difficulty Questionnaire, *LR* Lack of Readiness, *LI* Lack of Information, *II* Inconsistent Information, *PFC* Problem-focused coping strategies, *EFC* Emotion-focused coping strategies, *DC* Dysfunctional coping strategies

*p < 0.05

### Factors associated with career indecision

Linear regression analyses of the factors associated with career indecision are presented in Table 3. The results that CDDQ scale score was the dependent variable showed that in Block 1, experience in a career development program was significantly positively associated with career indecision (β = − 0.19, *p* < 0.05). When psychological health status was added in Block 2, experience in a career development program (β = − 0.17, *p* < 0.05) and psychological health status (β = 0.25, *p* < 0.05) were significantly associated with career indecision. Psychological health explained 6.0% of the variance of career indecision. In Block 3, PFC was negatively associated with career indecision, with a standardized coefficient of − 0.14 (*p* < 0.05). DC was significantly and positively associated with career indecision (β = 0.25, *p* < 0.05). However, EFC was not significantly associated with career indecision. Coping strategies explained 7.7% of the variance of career indecision. Hypothesis 1 was partially confirmed, as the association between adaptive coping strategies and career indecision was significant, but only for PFC. Hypothesis 2 and hypothesis 3 were confirmed. The results that LR, LI, and II score were the dependent variable were also displayed in Table 3.

**Table 3:** Linear regression analyses of the factors associated with career indecision (*n* = 359) ^a^

Dependent variables	Independent variables	Block 1 (β)	Block 2 (β)	Block 3 (β)
CDDQ	Experience of career development program-Yes	−0.19*	− 0.17*	− 0.16*
	Psychological problem-Yes		0.25*	0.20*
	PFC			−0.14*
	EFC			−0.08
	DC			0.25*
	F	4.43*	7.94*	9.45*
	*R*^2^	0.059	0.119	0.196
	△*R*^2^	0.059	0.060	0.077
LR	Experience of career development program-Yes	−0.15*	− 0.12*	− 0.12*
	Psychological problem-Yes		0.25*	0.21*
	PFC			−0.08
	EFC			−0.07
	DC			0.24*
	F	3.45*	7.02*	7.49*
	*R*^2^	0.047	0.107	0.162
	△*R*^2^	0.047	0.060	0.055
LI	Experience of career development program-Yes	−0.24*	− 0.21*	−0.20*
	Psychological problem-Yes		0.25*	0.21*
	PFC			−0.18*
	EFC			−0.09
	DC			0.17*
	F	5.46*	8.84*	9.46*
	*R*^2^	0.072	0.131	0.196
	△*R*^2^	0.072	0.059	0.065
II	Experience of career development program-Yes	−0.17*	−0.15*	−0.15*
	Psychological problem-Yes		0.21*	0.16*
	PFC			−0.14*
	EFC			−0.06
	DC			0.26*
	F	2.91*	5.25*	7.36*
	*R*^2^	0.040	0.082	0.159
	△*R*^2^	0.040	0.042	0.077

^a^ Basic characteristics, including age, sex, registered residence, and study year, were controlled for but not shown. *p < 0.05; *β* standardized regression coefficient, *CDDQ* Career Decision-making Difficulty Questionnaire, *LR* Lack of Readiness, *LI* Lack of Information, *II* Inconsistent Information, *PFC* Problem-focused coping strategies, *EFC* Emotion-focused coping strategies, *DC* Dysfunctional coping strategies

## Discussion

The difficulties regarding LR most frequently occurred in medical students when making career decisions, with the highest score of 2.48 ± 0.58. This result was consistent with previous studies on Chinese nurse students [25], American university students [2], and Romanian high school students [26]. Since these difficulties could inhibit the beginning of career decision-making processes, if students do not deal with these difficulties in the earlier stages of their education, there may be more negative career-related outcomes before graduation [9, 26]. In medical education, earlier career awareness was also prompted. Shortening the medical career pathway and refining career intentions were suggested in the foundation years [5, 27].

The most common coping strategy applied by medical students was adaptive coping strategies, PFC. Similar results were found in studies from India and Pakistan, where medical students utilized the active coping strategies more frequently when dealing with various stressors [28, 29]. The present study also showed that being at a higher level of career indecision is negatively associated with PFC (β = − 0.14, *p* < 0.05). These results are consistent with previous findings among college students and young adults [1, 18]. Overall, these findings could be explained by that adaptive coping strategies used by medical students could maintain their confidence, help them to focus on high career aspirations, and contribute to their career decision-making processes [30, 31].

For the maladaptive coping strategies, DC showed a significantly positive association with career indecision among medical students, and the coefficient was the highest (β = 0.25, *p* < 0.05). Previous studies have also found that people using nonproductive coping strategies, such as escape, helplessness, and isolation, can be maladaptive and predispose the individual to experiencing career indecision [1, 31]. Individuals with coping strategies that encourages avoidance will be inclined to defer negative outcomes or make hasty and irrational decisions. Furthermore, these coping mechanisms may place medical students at risk of distress, and the use of these strategies may become habitual [32]. In light of the above results, medical educators and career counselors should focus on helping medical students reduce the use of maladaptive coping strategies rather than solely to enhance the use of the adaptive strategies, such as problem-focused [1, 31]. Specifically, there are also cognitive techniques, such as Mindfulness-Based Interventions (MBIs), to reduce dysfunctional coping and promote adaptive coping for medical students [33, 34].

Among the associated factors in this study, career indecision was positively associated with psychological distress (*p* < 0.05). This result is in line with other research reporting that psychological distress, depression, and anxiety were related to career indecision among college students and emerging adults [35–38]. Multon et al. also found that psychological distress influences career counseling outcomes [39]. These findings could be explained by the positive psychological framework, which postulates that positive psychological functioning enables individuals to achieve successful career decision-making [9]. Furthermore, high prevalence (28%) of psychological distress problem was found among medica students in our study. The psychological problems among medical students are of growing concern around the world [28, 29]. Hence, career indecision and psychological distress are intertwined and should be treated holistically when conducting the career intervention for students [35, 36, 39].

Consistent with previous studies [40, 41], it is not surprising to find that medical students participating in a career development program had lower career indecision levels. The career development skills obtained from the program could increase student confidence in career exploration and decision making [40]. However, only 36.5% of the study participants had this experience. The value of offering a structured program to help medical students with making decisions about their career has been recognized by medical schools, and attempts have been made to structure and formalize career advice by including it in accreditation standards of both America and Canada [42–44].

There are limitations to this study that should be acknowledged. One limitation of the present study is that it was conducted among senior medical students. Ideally, providing advice on career decision-making processes would begin the first week of medical school and continue throughout the education program [43]. Future studies are needed to replicate the findings in medical students from all study years. Another limitation is that this study only included Chinese medical students, and the findings may be influenced by cultural factors, as previous studies have indicated that the effectiveness of coping strategies should be considered within the specific cultural context [1, 18]. Future studies should be conducted in other countries to compare the results of different cultural contexts. Additionally, the cross-sectional design prevented the study from establishing a causal relation between career indecision and the associated factors that were examined. It should also be suggested that the factors that were associated with career indecision be addressed separately and in more depth in future research.

## Conclusions

The difficulties regarding LR frequently occurred in medical students when making career decisions. This implies that it is necessary to promote earlier career awareness to prevent career indecision among medical students. Positive associations were found between psychological distress, dysfunctional coping strategies, and career indecision. Medical students who adopted problem-focused coping strategies were found to have fewer career decision-making difficulties. These findings imply that specifically, during the career intervention process for medical students, improving psychological health should be considered as a motivating factor for medica students with career indecision. Additionally, when helping medical students with career indecision, the coping style applied by individuals should be also addresses. And cognitive techniques that reduce the use of maladaptive coping strategies and enhance the use of the adaptive ones should be adopted.

## Data Availability

The dataset used and analysed during the current study available from the corresponding author on reasonable request.

## Abbreviations

CDDQ: Career Decision-making Difficulty Questionnaire
LR: Lack of Readiness
LI: Lack of Information
II: Inconsistent Information
Brief COPE: Brief Coping Orientation to Problems Experienced
GHQ-12: 12-item General Health Questionnaire
PFC: Problem-focused coping strategies
EFC: Emotion-focused coping strategies
DC: Dysfunctional coping strategies
VIF: Variance inflation factor
MBIs: Mindfulness-Based Interventions
